# Preface

**DOI:** 10.1093/jxb/erv153

**Published:** 2015-04-20

**Authors:** Ian Dodd, Howard Griffiths, Robert Sharp, Mary Traynor, Jianhua Zhang

**Affiliations:** Lancaster University, UK; Cambridge University, UK; University of Missouri, USA; Journal of Experimental Botany; The Chinese University of Hong Kong

Since the 1970s, **Bill Davies**’ research has revolutionized our understanding of how plants sense and respond to changes in the environment. Although a UK native, Bill pursued his graduate and postdoctoral studies in the USA where he gained formative training in plant water relations with Theodore Kozlowski at the University of Wisconsin, Madison, and Paul Kramer at Duke University. Bill returned to the UK in 1975 to establish his own programme at Lancaster University, and his laboratory rapidly established a highly influential presence in the field of plant environmental physiology. Early and continuing areas of focus included stomatal control of leaf gas exchange, including collaborative studies with Lancaster colleague **Terry Mansfield,** as well as leaf growth, with important contributions from **Liz Van Volkenburgh** (now at the University of Washington, USA) and **Gail Taylor** (now at the University of Southampton). Two of Bill’s first PhD students also began to delve into the under-explored ‘hidden half’ of the plant, including pioneering studies of root growth and osmotic adjustment in drying soil by **Oluwole Osonubi** (now at the University of Ibadan, Nigeria) and **Bob Sharp** (now at the University of Missouri, USA).

Starting from the mid-1980s, Bill’s laboratory showed that plants can sense gradual soil drying by regulating their water loss from the leaves. This ‘feed-forward’ response can occur before plants show any visible leaf wilting. **Jianhua Zhang** (now at the Chinese University of Hong Kong) joined the team in 1985 and found that a root-sourced chemical signal, later identified as ABA, was produced in the drying roots and transported to the shoots to regulate stomatal opening and leaf expansion. Following this discovery, a highly successful collaboration with **Francois Tardieu’s** group (then at INRA Versailles, now Montpellier) found that plants grown in the field under different soil conditions (drought, compaction) showed a unified relationship between xylem sap, ABA concentration, and stomatal conductance. This work paved the way for the development of a widely-applied model of the regulation of stomatal conductance (the Tardieu–Davies model), which is updated in this issue (Tardieu *et al.*, 2015).

Such root-to-shoot signalling (of soil drying and other environmental stresses) attracted much attention by the research community, culminating in a very-well-attended meeting held in Lancaster in 1989, which gave rise to the famous ‘yellow book’ (Davies and Jeffcoat, 1990), a firm favourite of the many PhD students that joined the Davies laboratory in the 1990s. Furthermore, a seminal review summarized much of the information on root-to-shoot signalling of drying soil (Davies and Zhang, 1991), and has attracted well over 1000 journal citations and has been documented in all the plant physiology textbooks since the 1990s.

The 1990s were a busy time in Laboratory B14 (shared between Bill’s and Terry Mansfield’s research groups), with research staff, PhD students, and visiting researchers arriving from around the world (including Australia, Germany, Malaysia, Mexico, Sri Lanka, and Sweden). Technological drawcards included a high throughput radioimmunoassay for ABA (adopted from **Steve Quarrie’s** laboratory), cellular pressure probes for measuring turgor, and Ingestad units for tightly controlling plant nutrition. Important issues that occupied the attention of the group during this time included the relative importance of ABA flux and concentration in regulating stomatal responses, the role of ABA metabolism (with key contributions from **David Gowing** and **Carlos Trejo**), and whether there was sufficient ABA in xylem sap to effect stomatal closure. Seminal work by **Sally Wilkinson** in collaboration with **Wolfram Hartung** (Universität Würzburg) established that xylem sap pH was critical in regulating the access of ABA to sites of action (guard cells and expanding cells) in the leaf. Thus, it seemed there was always sufficient ABA in the xylem sap to close the stomata: the issue was whether xylem sap pH favoured the compartmentation of ABA into mesophyll cells (with acidic xylem pH) or into the guard cell apoplast (with alkaline pH), thereby effecting stomatal closure (Wilkinson and Davies, 1997).

Unlike many discoveries in the natural sciences, Bill Davies didn’t stop here with fundamental scientific discoveries. He co-ordinated several groups worldwide and started to develop an irrigation method by making use of the plant long-distance signalling mechanism. The so-called Partial Root-zone Drying/Irrigation (PRD or PRI) has been successfully developed and applied in several countries, including Australia (in collaboration with **Brian Loveys,** CSIRO Adelaide), California, Turkey, and China. The essence of PRD is to always keep part of the root system in drying soil during irrigation, and the drying and wetting zones can be alternated in subsequent irrigation. Such practice has achieved huge success. **Jianhua Zhang** together with his long-term collaborator **Kang Shaozhong** at the China Agricultural University have successfully developed and promoted this irrigation method in Northwest China where so-called oasis agriculture almost totally relies on irrigation (Kang and Zhang, 2004). On average, 30% of the irrigated water can be saved while the yield quantity and quality of many crops are maintained. Such efforts have largely deterred the rapid desertification and shrinking of oases that were occurring just a few years ago.

Scientific research into, and the commercial introduction of, PRD provided the impetus for large European (the EU IRRISPLIT project) and national (DEFRA-funded) projects. **Mark Bacon** played a leading role in managing these projects and securing significant commercial interest in this irrigation technique, and the plant sciences more generally, under the auspices of agronomy training programmes developed for the Waitrose supply chain. Commercial implementation of PRD stimulated some very practical questions concerning the nature of root-to-shoot signalling under spatially and temporally varying soil moisture, which provided a significant research strand for **Ian Dodd,** who re-joined the laboratory in the 2000s. His meta-analysis of the literature demonstrated that PRD often out-yielded conventional deficit irrigation when the same irrigation volumes were applied using both techniques (Dodd, 2009), and differences in root-to-shoot signalling appear partially responsible.

Nevertheless, Bill’s group continued to search for additional mechanisms by which to manipulate the growth of plants in drying soil. Observations that the plant hormone ethylene may be an important signal and that transgenic attenuation of ethylene signalling maintained leaf growth in drying soil (Sobeih *et al.*, 2004) stimulated the development of non-GM methods of producing the same effect. A series of Royal Society-sponsored research grants allowed **Andrey Belimov** (of the All-Russia Research Institute of Agricultural Microbiology, St Petersburg) to investigate the potential of using ACC-deaminase containing rhizobacteria to attenuate root-to-shoot ACC signalling, thereby alleviating growth inhibition caused by drying soil. More recent work (led by **Sally Wilkinson**) has also revealed an important effect of ethylene on stomatal functioning, which appears to involve an ethylene-stimulated blocking of the effects of ABA.

Despite his horticultural roots (Bill’s father grew lettuces) and significant work on horticultural crops, Bill’s recent research efforts have focused on whether phytohormone signalling can play a role in regulating yield-determining processes in arable (both food and fuel) crops. This has been pursued by evaluating the drought-stress responses of diverse germplasm with European projects (EU DROPS co-ordinated by **Francois Tardieu** and EU WATBIO co-ordinated by **Gail Taylor**) and the International Wheat Yield Consortium directed by CIMMYT.

As of right now, many parts of the world (representing nearly 3 billion people) are suffering from a shortage of water. Agriculture consumes 70% of water resources available to humans and this has caused some serious environmental problems in arid or semiarid areas where irrigation is the only way to maintain a stable food output. In China, for example, well over 50% of its food production, distributed in the North China Plain and Northwest China, is under consistent threat of drought and water shortage (Zhang, 2011; Du *et al.*, 2014). This makes water shortage the number one threat to crop production in these areas and, consequently, research into methods of water-saving agriculture is essential for combating the challenge. It should be recognized that **Bill Davies** has played a leading role in such efforts. The significance of the work is obviously evidenced by the huge magnitude of water-saving agriculture and the sheer size of the number of farmers who can benefit worldwide.

Bill has also had a lasting impact on the publishing and academic science communication world. He was one of the founders of the Plant Environmental Physiology group, jointly constituted between the SEB and BES. He played a particularly important role in maintaining the profile and outputs of that group by organizing meetings and symposia, several culminating in Special Issues of the *Journal of Experimental Botany*. He was also a founding editor of the ‘Environmental Plant Biology’ series published originally by BIOS in conjunction with the SEB, a highly successful series which included an inaugural volume on ABA, followed closely by titles on Carbon Partitioning, Water Deficits, and Stable Isotopes.

Bill was Editor-in-Chief of the *Journal of Experimental Botany* from 1995 to 2007. During this time the Journal was the first plant journal to move from print only to full electronic and print publication, and the first to introduce a new and unique Open Access publication model that is central to the success of the journal to date. Bill also introduced the publication of a series of highly cited and well-respected Special Issues, of which this issue forms part.

This special issue, originating from the ‘Roots to Global Food Security’ meeting at the SEB Annual Meeting in Manchester (2–4 July 2014), not only showcases fundamental science in root growth regulation, root-to-shoot signalling, and crop management, but also demonstrates its importance in securing global food supplies. Many of the authors of the articles collected herein conducted their PhD studies or research within Bill Davies’ group, and a significant feature of this group is the number of PhD graduates who have gone on to forge significant scientific and publishing careers of their own throughout the world. This meeting provided not only an opportunity to discuss issues of scientific importance to Bill, but, once again, to draw together colleagues who have benefited from his friendship and guidance over the years.

We hope you enjoy this issue!
